# Overview of Stabilizing Ligands for Biocompatible Quantum Dot Nanocrystals

**DOI:** 10.3390/s111211036

**Published:** 2011-11-28

**Authors:** Yanjie Zhang, Aaron Clapp

**Affiliations:** Department of Chemical and Biological Engineering, Iowa State University, Ames, IA50011, USA; E-Mail: yanjiez@iastate.edu

**Keywords:** quantum dots, ligand, biocompatible

## Abstract

Luminescent colloidal quantum dots (QDs) possess numerous advantages as fluorophores in biological applications. However, a principal challenge is how to retain the desirable optical properties of quantum dots in aqueous media while maintaining biocompatibility. Because QD photophysical properties are directly related to surface states, it is critical to control the surface chemistry that renders QDs biocompatible while maintaining electronic passivation. For more than a decade, investigators have used diverse strategies for altering the QD surface. This review summarizes the most successful approaches for preparing biocompatible QDs using various chemical ligands.

## 1. Introduction

Due to their sensitivity, reliability, and rapid response, fluorescence-based techniques are now vital in biological applications including *in vivo* and *in vitro* cellular targeting and imaging, molecular imaging, and multiplexed analyses. These applications require an appropriate fluorescent probe having specific chemical and optical properties. Organic and genetically-encoded fluorophores generally have narrow absorption, broad emission, and are vulnerable to continuous irradiation by the excitation source which limits their usefulness in some applications such as multiplexed measurements, long-term imaging, and single molecule imaging, among others [1,2]. While fluorescent dyes and proteins have been applied broadly to problems in biological sciences, some of their inherent weaknesses have significantly limited the potential of related fluorescence techniques. Quantum dots (QDs) are appealing alternatives to conventional fluorophores due to their superior optical properties and have the potential to meet some of these outstanding challenges in biotechnology. However, the surface chemistry of QDs and the presence of the surface trap sites usually tend to deteriorate their properties in terms of quantum yield and stability. This aspect of QD synthesis remains a considerable challenge for researchers. In this review, we examine some of the most successful methods for rendering QDs biocompatible with the goal of inspiring new and related methods that realize the vast potential of these fluorophores.

**Figure 1.**
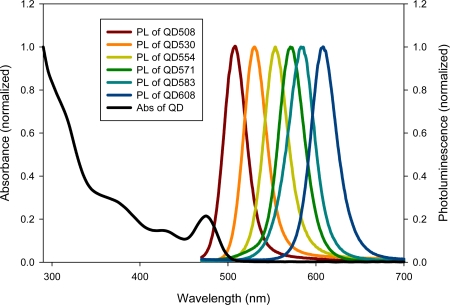
Absorption and emission of six different quantum dot populations.

The physical size of colloidal QDs is usually the range of 2–15 nm in diameter where the energy levels of the excited state electron-hole pair (or “exciton”) are quantized rather than continuous. Within this nanocrystal size range, which is on the order of or smaller than the exciton Bohr radius, QDs cease to behave as bulk materials due to confinement of their charge carriers (electrons and holes) in three spatial dimensions. This endows QDs with size, shape, and composition-dependent physical/optical properties that are readily tunable during synthesis. Unlike conventional fluorophores, QDs have broad absorption spectra (QDs of multiple colors can be excited by a monochromatic source), narrow, symmetric, and size-tunable emission spectra (where it is relatively easy to distinguish one QD population from another, as shown in Figure 1); high resistance to physical and chemical degradation (suitable for long-term imaging); high extinction coefficients and quantum yields (sufficient for single molecule measurements); and long fluorescence lifetimes (on the order of 10 ns, useful in time-gated experiments) [2,3]. Because of their appealing and tailorable properties, they have been widely used in a diverse range of fields from clinical diagnostics [4–6] to photovoltaics [7,8].

QDs are generally synthesized using an organometallic approach in which they are stabilized by hydrophobic surfactants and are therefore initially soluble in non-polar media. It is critical to render QDs water-soluble through modification of their surface in preparation for biological applications. An ideal water-soluble ligand should meet the following requirements: (1) provide QDs stability and solubility in biological buffers; (2) maintain a high resistance to photobleaching and other photophysical properties in aqueous media; (3) have functional groups which are able to conjugate to biomolecules; (4) minimize overall hydrodynamic size. The stability of QDs in water can be obtained by either a complete ligand exchange procedure, or through steric stabilization where the native hydrophobic surface is coated with amphiphilic molecules and/or polymers [2]. Both of these methods have their own advantages where the ultimate choice largely depends on the specific application and requirements of the nanocrystals. Small molecules carrying a net charge tend to contribute to aggregation in buffers of high ionic strength and exhibit variable stability with changes in pH. Additionally, electrostatically stabilized QDs can increase the possibility of non-specific binding in certain biological environments [2]. Although coating with polymers can effectively mitigate these problems, for example by creating a steric barrier around the nanocrystal, the effective hydrodynamic diameter of these QDs can increase three to four fold [9,10] which may preclude uptake into cells via endocytosis or disrupt signal transduction in sensing applications that utilize distance-dependent fluorescence resonance energy transfer (FRET).

Here we review recent progress for preparing biocompatible QDs including aqueous phase synthetic methods, cap exchange procedures that completely replace hydrophobic ligands with hydrophilic species, encapsulating QDs with amphiphiles, and coating QDs with solid shell layers.

## 2. Aqueous Synthesis of Quantum Dots

**Figure 2.**
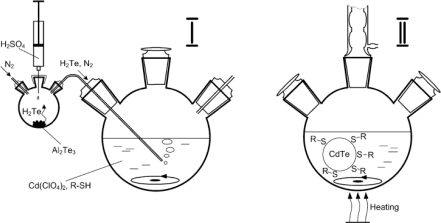
Schematic of CdTe synthesis using aqueous method [16]. Copyright 2002 American Chemical Society.

Other than the organometallic approach, QDs can be synthesized through a more direct approach toward water-solubility by using phosphates [11] or thiols [12–15] acting as stabilizing agents in aqueous media. The ligands used during synthesis become the eventual biocompatible surface ligands and do not require an exchange procedure. Figure 2 illustrates a typical aqueous synthesis of CdTe [16] which is completed in two distinct stages. Cd and Te precursors are mixed in stage I in the presence of a thiol stabilizer. CdTe nanocrystals nucleate and grow by reflux in stage II. Gaponik and co-workers [16] investigated the effect of thiol-capping of CdTe QDs and found that each ligand has a unique effect on the overall performance of QDs (Table 1).

The growth rate and surface charge of the nanocrystals depended on the choice of stabilizers. They had difficulty in achieving bright QDs that also exhibit long-term colloidal stability. However, the quantum yield (QY) of these thiol-stablized QDs can be improved by various post-synthesis treatments such as photochemical etching [16], long term exposure to illumination by a mercury discharge lamp [17] (photo annealing), and size-selective precipitation [13].

Glutathione (GSH), a thiol-containing tripeptide, which is known to detoxify heavy metals in plant cells, is able to provide improved biocompatible capping for QDs as compared with many other water-soluble ligands [18]. It has been used successfully to cap Au, ZnS, CdS, CdSe [19–21] and CdTe nanoparticles, but appears to work best with CdTe in terms of promoting high photoluminescence [22]. The pH of the solution containing the Cd^2+^-GSH precursors and the molar ratio of Cd^2+^-GSH to NaHTe (Te precursor) were found to have a strong influence on the QY of CdTe QDs. Higher molar ratios resulted in better passivation of the surface states on CdTe which led to higher QY. This effect saturated with complete surface passivation by Cd^2+^-GSH.

Most reported aqueous synthesis methods have focused on II–VI group nanocrystals, while there are few reports regarding the synthesis of III–V QDs in water, save the work of Qian’s group [23]. They prepared InP QDs in aqueous ammonia in the presence of potassium stearate. However, this hydrothermal method can only produce nanocrystals in the form of secondary particles [24].

## 3. Direct Ligand Exchange

QDs synthesized via the organometallic method require an extra processing step to achieve biocompatibility. The direct ligand exchange is accomplished by replacing QDs’ native hydrophobic ligands with hydrophilic molecules that have higher affinity for the QD surface. The most popular anchoring molecules utilize thiol groups. Others including amines, phosphonic acids, and carboxylic acids have also been widely used [25–28].

### 3.1. Monodentate Thiols

In 1998, Nie’s group [29] developed one of the first protocols for generating hydrophilic QDs by coating CdSe/ZnS core-shell QDs with mercaptoacetic acid which exploits the affinity of monothiols for the ZnS shell. Since then, many other monothiol ligands have been used to provide QDs water-solubility such as mercaptopropionic acid (MPA) [30–32], mercaptoundecanoic (MUA) [33], 4-mercaptobenzoic acid [34], thiol-derivatized sugar [35], thiolated derivative of diethyleneglycol [36], dendrons [37,38], cystamine [2], cysteine [39] and related residues [40]. This approach continues to be a popular method due to the ease of processing and the commercial availability of candidate ligands.

Due to the dynamic binding interactions between the thiol and ZnS, QDs coated with monothiol ligands tend to have shorter shelf lives [41]. Also, the pH of the solution is found to be responsible for the thiolate ligand stability. Peng’s group [42] used a pseudo steady-state titration method to investigate the stability of colloidal nanocrystals coated with thiolate ligands upon pH change. They found that as pH of the solution fell into a relatively low range (2–7), the thiolate ligand became protonated and dissociated from the nanocrystal surface. This effect was confirmed as the main reason for the precipitation of the nanocrystals. This dissociation process was found to be reversible and the equilibrium pH value was size dependent. Similarly, the luminescence QY tends to suffer with these samples presumably due to the instability of the ligand and reduced surface coverage over time. The quenching efficiency was found to depend strongly on both the size and charge of the thiol [43]. Several groups have found that an appropriate ligand exchange method is crucial for preserving colloidal stability and brightness. Pong *et al.* [44] discovered that the bond strength between a monothiol and ZnS can be increased by removing thiolic hydrogen during the adsorption process. Wang *et al.* reported [45] that the photoluminescence can be maintained if the ligand exchange step was eliminated as a separate step. Alternatively, they developed a new strategy, *in situ* shell formation and ligand capping, which allowed the core CdSe to be overcoated by a ZnS shell and the water-soluble ligand MPA in a single step. They further found the QY of synthesized CdSe/ZnS core-shell QDs capped with MPA was comparable to that of trioctylphosphine oxide (TOPO)-capped QDs while the QY of MPA-capped CdSe-ZnS QDs using ligand exchange method was five-fold lower. Tamang *et al.* [46] found that pH played an important role in increasing stability of InP/ZnS QDs during an aqueous phase transfer procedure. The thiol group of a water-soluble ligand is deprotonated at high pH, and thiolate has been shown to bind the QDs surface more strongly than thiol [47]. They also discovered that the QY could be improved by adding an appropriate reducing agent.

The QD surface states have a significant effect on the quantum yield. Jeong *et al.* [48] found that it was the thiolate, not thiol, that had a time-, concentration-, and pH-dependent effect on QD surface states. They proved that the thiol addition could better passivate the electron trap sites and therefore increased the QY at low thiol concentration, while at high thiol concentration new hole traps would be created and QY decreased.

A reduction in the luminescent QY mediated by thiol binding can be mitigated by either crosslinking or adding layers to the surface [49]. Chan’s group [50] first coated hydrophobic QDs with MPA and then crosslinked these ligands with lysine or diaminopimelic acid in the presence of dicyclohexylcarbodiimide. They later examined stability of these QDs in various biological conditions in order to optimize their behavior for various applications. This cost-effective, simple method was able to synthesize highly stable water-soluble QDs which maintained all of the hydrophobic QD optical properties. However, due to the relatively thick coating layer, the size was twice as big as the TOPO coated QDs.

**Figure 3.**
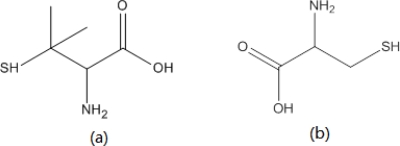
Chemical structures of **(a)** d-pencillamine and **(b)** cysteine.

Zwitterionic monothiols, possessing positive and negative charges simultaneously, have the potential to minimize hydrodynamic particle size and reduce nonspecific binding. Bawendi’s group [51] found that zwitterionic or net neutral charged organic coatings prevented the binding of QDs to serum proteins. They further found that QDs having a hydrodynamic diameter below 5.5 nm can be efficiently removed from the body by renal clearance. They compared four different coatings including dihydrolipoic acid (DHLA, anionic), cysteamine (cationic), cysteine (zwitterionic), and DHLA-polyethylene glycol (DHLA-PEG, neutral). Of these, only cysteine and DHLA-PEG coated QDs were able to prevent the adsorption of serum proteins. However, the effective size of the DHLA-PEG coated QDs was too large to be eliminated via renal clearance. Although they showed promising results, the main drawback of cysteine-coated QDs was their instability. These samples typically precipitated after 24 hours of storage at 4 °C [39]. Breus *et al.* [52] used zwitterionic d-penicillamine (DPA) as a stabilizing ligand for QDs and showed that they are far more stable than cysteine-coated QDs. The chemical structures of DPA and cysteine are shown in Figure 3. These DPA-coated QDs were shown to be stable over a pH range of 5–9 and exhibited excellent chemical stability even in strongly oxidizing environments. They hypothesized that the superior stability results from the reduced interaction between DPA ligands.

### 3.2. Bidentate Thiols

**Figure 4.**
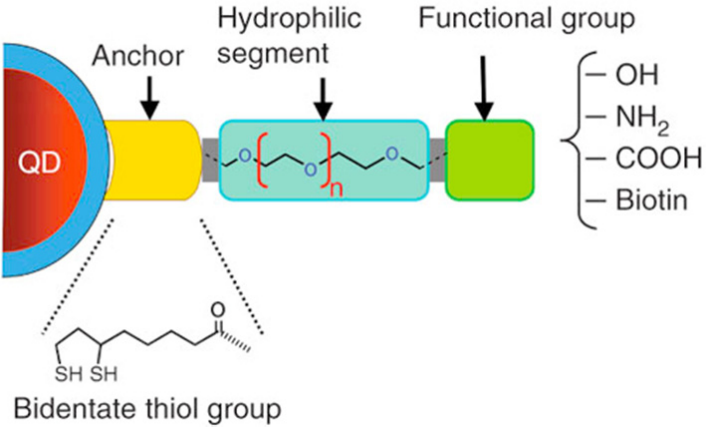
Modular design of QD hydrophilic ligands with different terminal functional groups based on DHLA-PEG [55]. Copyright 2007 American Chemical Society.

Recognizing the poor inherent stability of monothiol-coated QDs, Mattoussi *et al.* [53] overcoated QDs with negatively charged dihydrolipoic acid (DHLA) to render them water-soluble and promote attachment of engineered recombinant proteins through electrostatic attraction. These QDs demonstrated a dramatically improved shelf life of up to two years with proper storage. Nonetheless, DHLA is not able to preserve the high luminescence of native QDs presumably due to its lower density on the nanocrystal surface. Moreover, this negatively charged capping ligand is only stable in basic conditions (pH ≥ 7), and may induce non-specific binding to positively charged proteins in cellular applications. To circumvent this, the carboxylated DHLA-coated QDs can be further modified to have various functional groups which can serve different purposes. As Figure 4 shows, Susumu *et al.* [54,55] developed a new class of water-soluble ligands consisting of DHLA and PEG. The appended functional groups (hydroxyl, carboxylic acid, amino, and biotin) are readily able to conjugate with biomolecules. In addition to DHLA [56–58] and modified DHLA [59–63], other bidentate thiols such as dithiothreitol (DTT) [64], and thiol-modified β-cyclodextrin [65] have been successfully used as QD ligands, however none of these appears capable of achieving high luminescence and small size simultaneously.

**Figure 5.**
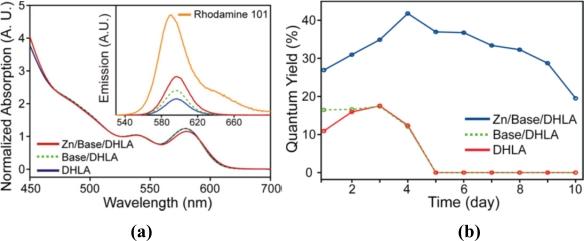
**(a)** Normalized absorption spectra of water-soluble CdSe/ZnS using different cap exchange methods. Inset: Emission spectra of same QDs and Rhodamine 101, excited at 525 nm. **(b)** Quantum yield of water-soluble CdSe/ZnS using different cap exchange methods over time. Zero quantum yield indicates the precipitation of the samples [66]. Copyright 2011 American Chemical Society.

Liu *et al.* [66] recently found that the quantum yield of DHLA-coated QDs can be increased twice during the ligand exchange process with the presence of zinc and base. Zn(DHLA)_2_ formed by metalation of zinc and DHLA and reacted with tetradecylphosphonic acid (TDPA) which initially capped hydrophobic QDs. They believed that this process was able to avoid the etching of the QD shell during cap exchange. They compared the quantum yield of QDs capped using different methods (with or without zinc/base) and found that the QY was improved with the help of zinc and base (Figure 5).

Reiss’s group [67,68] designed a new family of ligands called carbondithioates, which have high resistance to photooxidation and adsorb strongly onto the QD surface. However, a new specific synthesis is required to prepare every new ligand. Dubois *et al.* [69] then developed a facile and effective way to prepare similar ligands called dithiocarbamates (DTCs) by simply mixing carbon disulfide with an appropriate amine precursor. These DTC-coated QDs lost only 15% of their initial fluorescence after 500 consecutive illuminations at 350 nm (having a duration of 4 s each), where their absorption profiles were unchanged. Similar to effects seen with other thiolated capping molecules, the QY of these QDs was shown to drop substantially (∼40%) from its initial values. Based on similar chemistry, our own group [70] performed a ligand exchange procedure with DTCs in two phases rather than a single phase and found that the initial QY of hydrophobic QDs could be fully preserved or even enhanced if using high quality TOP/TOPO-capped CdSe/ZnS QDs. Another interesting finding is that these DTC-capped QDs can be either pH-insensitive or pH-sensitive based on the side chain group of the DTC ligand which may provide some advantage over traditional capping ligands.

### 3.3. Multidentate Ligands

Multidentate ligands offer QDs enhanced stability because of the increased number of binding sites between each ligand and the QD surface. Bawendi’s group [71] crosslinked alkyl phosphines, commonly used to stabilize hydrophobic QDs, with a crosslinker dissocyanatohexane to form polydentate ligands known as oligomeric phosphines (OPs). This new class of ligands consists of three components which are the inner phosphine layer (which passivate the QD surface), linking layer (which protect the QD), and an outer functionalized layer (which impart desirable chemical properties). The OPs displaying carboxylic acid groups were compared with MPA coatings on QDs with the former maintaining the initial QY and showing enhanced stability in various pH (ranging from 5.5 to 12) and in high ionic strength solutions. Wang *et al.* [72,73] passivated QDs with a multidentate diblock copolymer, poly(ethylene glycol-*b*-2-*N*,*N*-dimethylaminoethyl methacrylate) (PEG-*b*-PDMA). The pendent tertiary amine groups had proved to high affinity to the QD surface [74]. They found that the QY of QDs coated with 10k and 15k molecular weight polymer prepared by traditional solution polymerization were substantially higher than that of QDs coated with TOPO, while the presence of 35k molecular weight polymer synthesized by atom transfer radical polymerization (ATRP) decreased brightness of the QDs. They attributed this to two competing effects: enhanced surface passivation by the polymer and a parasitic effect caused by copper ion residual from the initiator during the ATRP synthesis.

**Figure 6.**
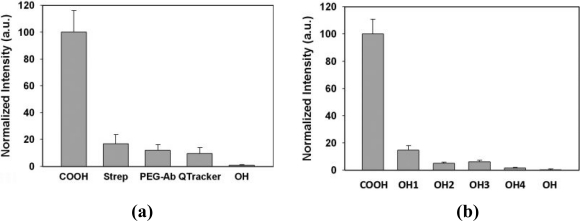
**(a)** Normalized photoluminescence intensity of QDs with different ligands measured by microplate assays. **(b)** Nonspecific cellular binding of QDs with different degree of hydroxylation [75]. Copyright 2008 American Chemical Society.

For certain biological applications, the QD capping ligands are required to minimize the nonspecific binding to the cell membrane or proteins. Nie’s group [75] prepared a new class of hydroxylated QDs, which initially have carboxyl groups, that are prepared through a hydroxylation and cross-linking procedure. These QDs had relatively small sizes (13–14 nm) as compared to other amphiphilic polymer-coated QDs (20–40 nm) [76–78]. They also evaluated their nonspecific binding properties with a series of different coatings including carboxylated, streptavidin-functionalized, and PEG-coated QDs; these results are shown in Figure 6. The hydroxylated QDs showed the lowest nonspecific cellular binding where a higher degree of hydroxylation helped to further reduce the nonspecific binding. To reduce the hydrodynamic size, they overcoated QDs with a linear polymer chain which had a mixed composition of thiols and amines [79]. This novel coating tightly wrapped around QD surface as “loops-and-trains” [73,74] where the size of QDs was as small as 4–7 nm in diameter. They further found that the number of available binding groups (thiols and amines) had strong influence on QD polydispersity, QY, and photostability. Increasing the number of binding groups offered better stability but sacrificed QY suggesting an optimal number of binding groups per QD.

Rather than using single DHLA as an anchoring group, Stewart *et al.* [80] synthesized a new set of multidentate PEG-based ligands which have two DHLA, or thioctic acid, molecules. These ligands improved QD stability to survive in conditions unfavorable to DHLA-capped QDs. They showed remarkably stability in various pH solutions ranging from 1.1 to 13.9 and extremely high salt conditions (2 M NaCl).

## 4. Indirect Surface Encapsulation

### 4.1. Silica Coatings

A silica shell is another desirable method for producing biocompatible nanocrystals due to the formation of a contiguous protective surface layer. QDs can acquire a silica layer either through direct ligand exchange [81–83] (surface silanization) or indirect encapsulation [84–88] (sol-gel process, microemulsion, micellization of siloxane surfactants). The deposited silica shell can be further modified to conjugate biomolecules using standard silane coupling chemistry that is already ubiquitous for modifying bulk glass surfaces such as microscope slides.

**Figure 7.**
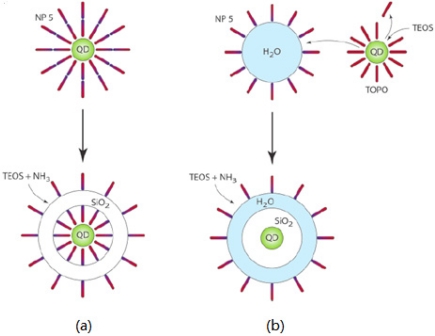
Scheme of two hypothetical mechanisms of silica growth on QDs. **(a)** Silica growth without ligand exchange. **(b)** Silica growth with ligand exchange and phase transfer [84]. Copyright 2005 American Chemical Society.

The sol-gel process was first developed by Stöber *et al.* [89] and requires water-soluble QDs as a starting material. Compared to the sol-gel process, the advantage of the microemulsion process is that it is able to overcoat hydrophobic QDs directly with a silica layer where the resulting nanoparticles have smoother surfaces and narrow size distributions [84]. Darbandi *et al.* [84] reported that the silica growth on QD surfaces involve two hypothetical mechanisms during the microemulsion process (Figure 7). The first possible mechanism is shown in Figure 7(a) where the surfactant forms an inverted bilayer around TOPO-coated QDs followed by deposition of a silica layer within the hydrophilic region carried out in the presence of an ammonia catalyst used to promote the polymerization of tetraethyl orthosilicate (TEOS). In the second hypothetical mechanism [Figure 7(b)], the TOPO ligand is replaced by TEOS and QDs are transferred to the aqueous phase followed by the polymerization of TEOS from the QD surface. The resulting nanoparticles were monodisperse and had high luminescence with a single QD in the center.

**Figure 8.**
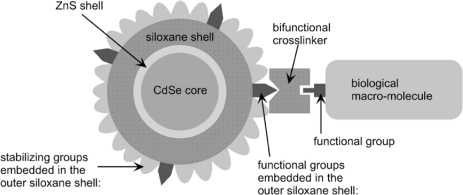
Modular design of silica-coated QDs with functional groups that is readily to crosslink with biological molecules [83] (phosphate, PEG, or ammonium groups on the outer siloxane surface act as stabilizing groups to maintain water solubility). Copyright 2002 Amercian Chemical Society.

Surface silanization is another way to form a silica shell around the QD by fully displacing the pre-existing hydrophobic ligands. In a typical process, TOPO-coated QDs are mixed with mercaptopropyltrimethoxysilane (MPTS) in alkaline methanol. The mercapto group is able to bind to the ZnS layer of QDs and thus replace the TOPO layer, followed by heating the solution to promote crosslinking of the silanol groups. The silica shell is later modified to have different functional group such as thiols, amines, or carboxyl groups to ensure covalent attachment to biomolecules (Figure 8) [83].

### 4.2. Amphiphilic Ligands

**Figure 9.**
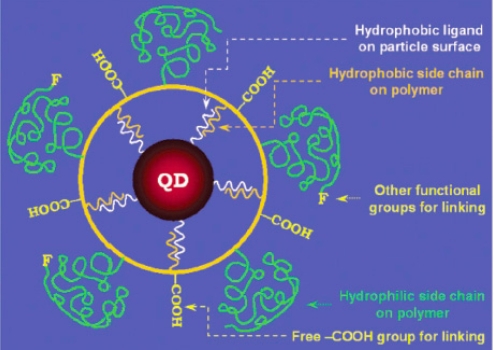
Scheme of amphiphilic polymer-coated QDs. The binding is through the hydrophobic attraction between the ligands that initially cap QDs and hydrophobic portion of the amphiphilic polymer. The outmost layer is modified with functional group for linking with other molecules [96]. Copyright 2007 American Chemical Society.

Unlike a direct ligand exchange process where hydrophilic ligands completely replace hydrophobic ligands, the method of encapsulating QDs with amphiphiles uses native nonpolar molecules as binding intermediates. The hydrophobic section of the amphiphiles intercalates the hydrophobic stabilizing agent such as TOPO while the hydrophilic portion offers aqueous solubility. Surfactants such as phospholipids [76,90,91], α-cyclodextrin [92,93], *n*-alcanoic acids [94], and cetyl-trimethylammonium bromide [95] have all been used to transfer nanoparticles into water. However, due to relatively weak hydrophobic interactions, they are normally not sufficiently stable when subjected to biologically relevant conditions [96]. The use of amphiphilic polymers can overcome this issue because a single polymer chain can possess multiple hydrophobic subunits which greatly enhances intercalation affinity with the hydrophobic ligands on the QD surface. Yu *et al.* [96] prepared the amphiphilic polymer poly(maleic anhydride-alt-1-octadecene) (PMAO)-PEG through the reaction between PMAO and primary amine-terminated PEG methyl ethers. Nonpolar QDs were then mixed with PMAO-PEG in chloroform and stirred overnight. The modular design of these amphiphilic polymer-coated QDs is shown in Figure 9. These PMAO-PEG-coated QDs were found to have the same optical properties, including QY, as hydrophobic QDs, and successfully recognized the cancer cells with the Her2 receptor. In this case, the binding between QDs and polymer ligands solely relies on hydrophobic attraction, however the length of the polymer improves this interaction substantially. The stability of amphiphilic polymer-coated QDs can be further improved by covalently crosslinking the outmost layer [78,97].

**Figure 10.**
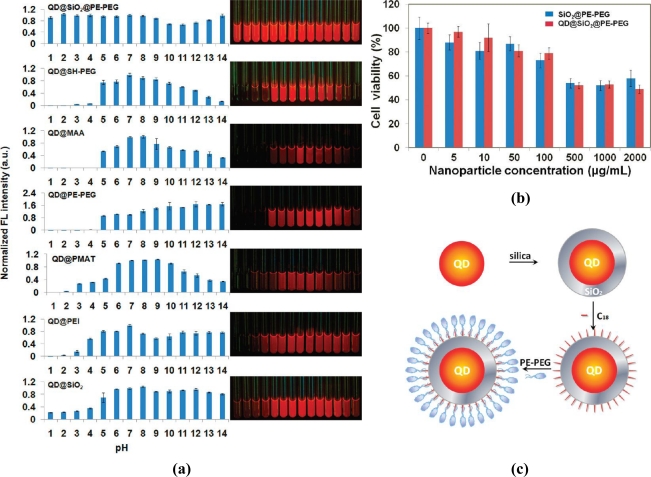
**(a)** Normalized fluorescence intensity of QDs capped with different coatings (SiO_2_-PE-PEG, thiolated PEG, mercaptoacetic acid (MAA), PE-PEG, poly(maleic anhydride alt-1-tetradecene) (PMAT), polyethylene imine (PEI) and SiO_2_) in various pH from 1 to 14 (tuned with HCl or NaOH). The photo on the right panel shows the corresponding florescence image under 365 nm hand-held UV lamp. **(b)** Scheme of incorporation QDs into SiO_2_-PE-PEG sphere. **(c)** Cell viability data of SiO_2_-PE-PEG-coated QDs and SiO_2_-PE-PEG nanoparticles without QDs doped inside [98]. Copyright 2010 American Chemical Society.

Gao’s group [98] found that capping QDs with silica and amphiphilic polymer together offered better passivation than capping with either one alone. Single hydrophobic CdSe/ZnS QDs were first incorporated into silica spheres via a reverse microemulsion, followed by surface modification with hydrophobic trimethoxy(octadecyl)silane (OTMS). The amiphiphilic polymer (1,2-distearoyl-*sn*-glycero-3-phosphoethanolamine-N-[carboxy(polyethylene glycol)-2000] (PE-PEG) was the attached to SiO_2_-OTMS-coated QDs through hydrophobic interactions [Figure 10(b)]. As Figure 10(a) shows, only QDs capped with SiO_2_ and PE-PEG showed similar brightness over pH 1–14. They also evaluated the cellular toxicity of these nanoparticles with and without embedded QDs and found that the toxicity is purely due to the exposure of bare nanoparticles, indicating QDs were adequately protected within the silica spheres with no appreciable leaching of heavy metals [Figure 10(c)].

## 5. Conjugating Quantum Dots to Biomolecules

To enable the use of QDs in a wider range of biological applications, all of the preceding methods require a route for attaching biomolecules stably onto the QD surface. A suitable ligand candidate should allow attachment of a diversity of biomolecules including nucleic acids, proteins (avidin/streptavidin, albumin, adaptor proteins, and antibodies), polysaccharides, and peptides. Biomolecules are commonly conjugated to the QD surface through the following approaches [99]: (1) covalent crosslinking: links carboxyl groups on the QD surface to amines by the use of 1-ethyl-3-(3-dimethylaminopropl) carbodiimide (EDC) [100] or 4-(N-maleimidomethyl)-cyclohexanecarboxylic acid N-hydroxysuccinimide ester (SMCC) [101], which crosslinks primary amine and sulfhydryl groups; (2) thiolated peptides (cysteine residues) can be directly conjugated to the QD surface through the affinity to ZnS shell layer [102,103]; (3) adsorption or non-convalent self-assembly using engineered proteins (e.g., a positively charged domain, His-tags) [104–106]. Owing to the large surface area-to-volume ratio, several biomolecules of varying types can be attached to a single QD. Each of these biomolecules provides a desired function which affords multi-functionality [6].

## 6. Summary

Biocompatible QDs are typically generated through either an organic synthetic route requiring subsequent ligand exchange or encapsulation, or a more direct aqueous synthetic route where the nanocrystals are inherently water-soluble. There are nearly countless variations of these methods reported in the literature where each protocol carries its own inherent advantages and disadvantages and must be considered in light of the eventual application. A ligand exchange procedure that uses small charged molecules can maintain minimal hydrodynamic diameters, but they are usually more susceptible to aggregation in biological buffers or solutions of high ionic strength. These compact capping ligands often fail to maintain suitably high luminescence without additional processing. Methods that improve the density of ligands on the QD surface while ensuring a strong binding interaction appear to have the most promise. Polymer and silica coatings are better able to isolate QDs from solution which results in improved colloidal stability, limited non-specific binding, and high QY. However, these characteristics are often achieved at the expense of compact size which can impede cellular mobility [107] and limit the ability to perform sensing via FRET. Although many excellent synthetic protocols already exist for creating biocompatible QDs, there is no ideal universal route, and each method must be considered against the requirements of the experiment. Continued research in this area is needed in order to realize the vast potential of QDs in biological applications, perhaps even resulting in human *in vivo* diagnostics and treatments.

## Figures and Tables

**Table 1.** Conditions for the aqueous synthesis of CdTe nanocrystals and their properties [16]. Copyright 2002 American Chemical Society.

**Stabilizer**	**pH used for the synthesis**	**Stability of CdTe QDs**	**Surface charge of CdTe QDs**	**Typical QY of as prepared CdTe QDs**
2-mercaptoethanol	11.2–11.8	stable	slightly negative in alkaline	<1%
1-thioglycerol	11.2–11.8	stable	slightly negative in alkaline	3%
mixture (1:1) of 1-thioglycerol and 2,3-dimercapto-1-propanol	11.2–11.8	moderate	slightly negative in alkaline	6%
thioglycolic acid (TGA)	11.2–11.8	stable	negative	10%
2-mercaptoethylamine (MA)	5.6–5.9	moderate	positive	10%
l-cysteine	11.2–11.8	moderate	negative or positive depending on the pH	10%
2-(dimethylamino)ethanethiol	5.0–6.0	moderate	positive	30%
